# *Pseudostellaria heterophylla* Extract Polysaccharide H-1-2 Suppresses Pancreatic Cancer by Inhibiting Hypoxia-Induced AG2

**DOI:** 10.1016/j.omto.2020.03.007

**Published:** 2020-03-31

**Authors:** Hongwei Sun, Keqing Shi, Kai Qi, Hongyu Kong, Qiye He, Mengtao Zhou

**Affiliations:** 1 Department of Hepatobiliary Surgery, Key Laboratory of Diagnosis and Treatment of Severe Hepato-Pancreatic Diseases of ZheJiang Province, The First Affiliated Hospital of Wenzhou Medical University, Wenzhou 325000, Zhejiang, China; 2 Shanghai Institute of Nutrition and Health, Chinese Academy of Sciences, Shanghai 200000, China; 3 Singlera Genomics, San Diego, CA 92037, USA; 4 Singlera Genomics (Shanghai), Shanghai 201203, China; 5 Precision Medical Center Laboratory, The First Affiliated Hospital of Wenzhou Medical University, Wenzhou 325000, China

**Keywords:** *Pseudostellaria heterophylla*, H-1-2, pancreatic cancer, anterior gradient 2, hypoxia

## Abstract

We aimed to examine the therapeutic potential of polysaccharide H-1-2, a bioactive component of *Pseudostellaria heterophylla*, against pancreatic cancer, as well as to demonstrate the underlying molecular mechanisms. Invasion and migration of pancreatic cells treated with H-1-2 were evaluated. A xenograft tumor mouse model was established to assess the effect of H-1-2 on tumor growth. Expression levels of hypoxic inducible factor-1α (HIF1α) and anterior gradient 2 (AGR2) were measured in pancreatic cells after H-1-2 treatment. Luciferase report and chromatin immunoprecipitation assays were conducted to investigate HIF1α regulation on AGR2. AGR2 expression was re-introduced into pancreatic cells to assess the role of AGR2 as a downstream effector of hypoxia after H-1-2 treatment. H-1-2 inhibited invasion and migration of pancreatic cancer cells, repressed xenograft pancreatic tumor growth, and increased survival of mice. H-1-2 repressed AGR2 expression in pancreatic cancer cells through the hypoxia response element (HRE) in its promoter region. Ectopic AGR2 expression partially negated the H-1-2 inhibitory effect on invasion and migration of pancreatic cells and on xenograft pancreatic tumors growth, and it also compromised the H-1-2 promotional effect on survival of mice. We conclude that H-1-2 suppresses pancreatic cancer by inhibiting hypoxia-induced AGR2 expression, supporting further investigation into its efficacy against pancreatic cancer in clinical settings.

## Graphical Abstract

**Figure:**
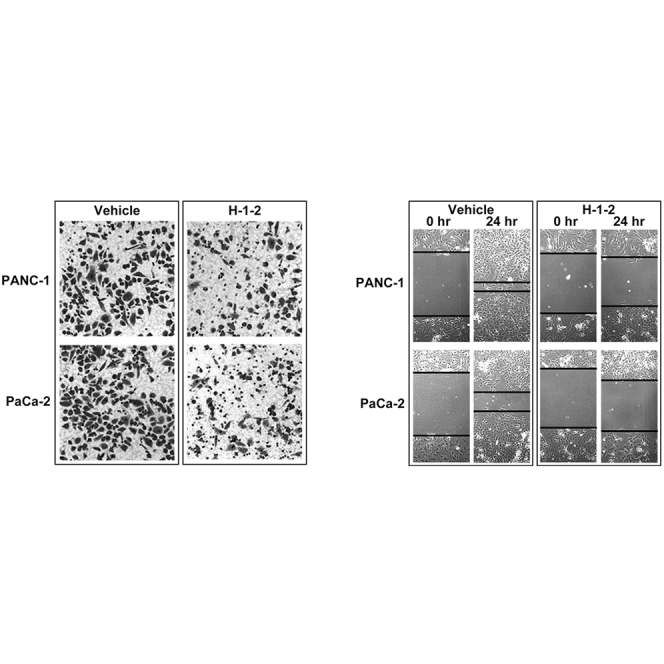

## Teaser

The therapeutic potential of polysaccharide H-1-2, a bioactive component of *Pseudostellaria heterophylla*, against pancreatic cancer was investigated in this study. We found that the polysaccharide H-1-2 suppresses pancreatic cancer by inhibiting hypoxia-induced AGR2 expression, supporting further investigation into its efficacy against pancreatic cancer in clinical settings.

## Introduction

Treatments for human cancer have advanced with the aid of recent progresses in cancer biology. However, pancreatic cancer fell drastically behind other types of tumors with regard to patient prognosis and survival.^1^ The overall 5-year survival rate of patients with pancreatic cancer is merely around 7%.^2^ Only about 10% of patients with pancreatic cancer are diagnosed when the disease is localized; therefore, less than 10% of diagnosed patients could be cured with surgical interventions. Furthermore, even among patients with localized cancer, the 5-year survival rate is only one in five. While multiple prominent morbidities associated with tumor, e.g., biliary sepsis, obstruction of gastric outlet and bile duct, tumor cachexia, and venous thromboembolism, are thought to contribute to the poor outcome, epidemiological investigations suggest that pancreatic tumor cells often mobilize and develop micrometastases at relatively early stages, thereby acquiring resistance to currently available adjuvant radiotherapy and/or chemotherapy.^3^ Hence, it is thought that pancreatic cancer fosters aggressive phenotypes at relatively early stages, different from other gastrointestinal cancers.

One essential player involved in the aggressive phenotype of pancreatic cancer is the microenvironment of the tumor tissues, which is characterized by extreme hypoxia.^3^ Hypoxic inducible factor-1 (HIF-1) is an essential player in the cellular adaption to hypoxic conditions, and it is enriched in most pancreatic tumor tissues. Prior investigations demonstrated that HIF-1 overexpression correlated with poor prognosis, but the underlying mechanisms remain unknown.^4^

*Pseudostellaria heterophylla* (*P. heterophylla*), also known as Prince Ginseng, has a history of application in Chinese traditional medicine.^5^ Composition analysis of *P. heterophylla* identified abundant fatty acids, amino acids, heterophyllin, polysaccharides, and diverse trace elements, among which the polysaccharides are considered to be the main constituent responsible for the beneficial effects of *P. heterophylla*.^6^ As early as decades ago, fractions from *P. heterophylla* have been proposed to stimulate the release of mitogenic and tumor necrosis factors.^7^^,^^8^ In particular, polysaccharide extracts from *P. heterophylla* were reported to exert immunostimulating activities against tumors.^9^ Furthermore, fractions of *P. heterophylla* that are enriched in polysaccharides could protect cardiomyocytes against cobalt chloride (CoCl_2_)-induced hypoxic injury,^10^ although the exact bioactive component responsible for the protection still remains unknown.

H-1-2, a novel homogeneous polysaccharide, was recently isolated from the polysaccharide fractions of the *P. heterophylla* extracts,^11^ which was later shown to inhibit hypoxia in human pancreatic β cells.^12^ These studies have prompted us to hypothesize that polysaccharide H-1-2 might exert the same anti-hypoxic activity in pancreatic cancer cells and suppress tumor progression. In addition, we sought to elucidate the downstream molecular mechanism responsible for the inhibitory effect of H-1-2 on pancreatic cancer.

## Results

### H-1-2 Inhibits Invasion and Migration of Pancreatic Cancer Cells *In Vitro*

**Figure 1:**
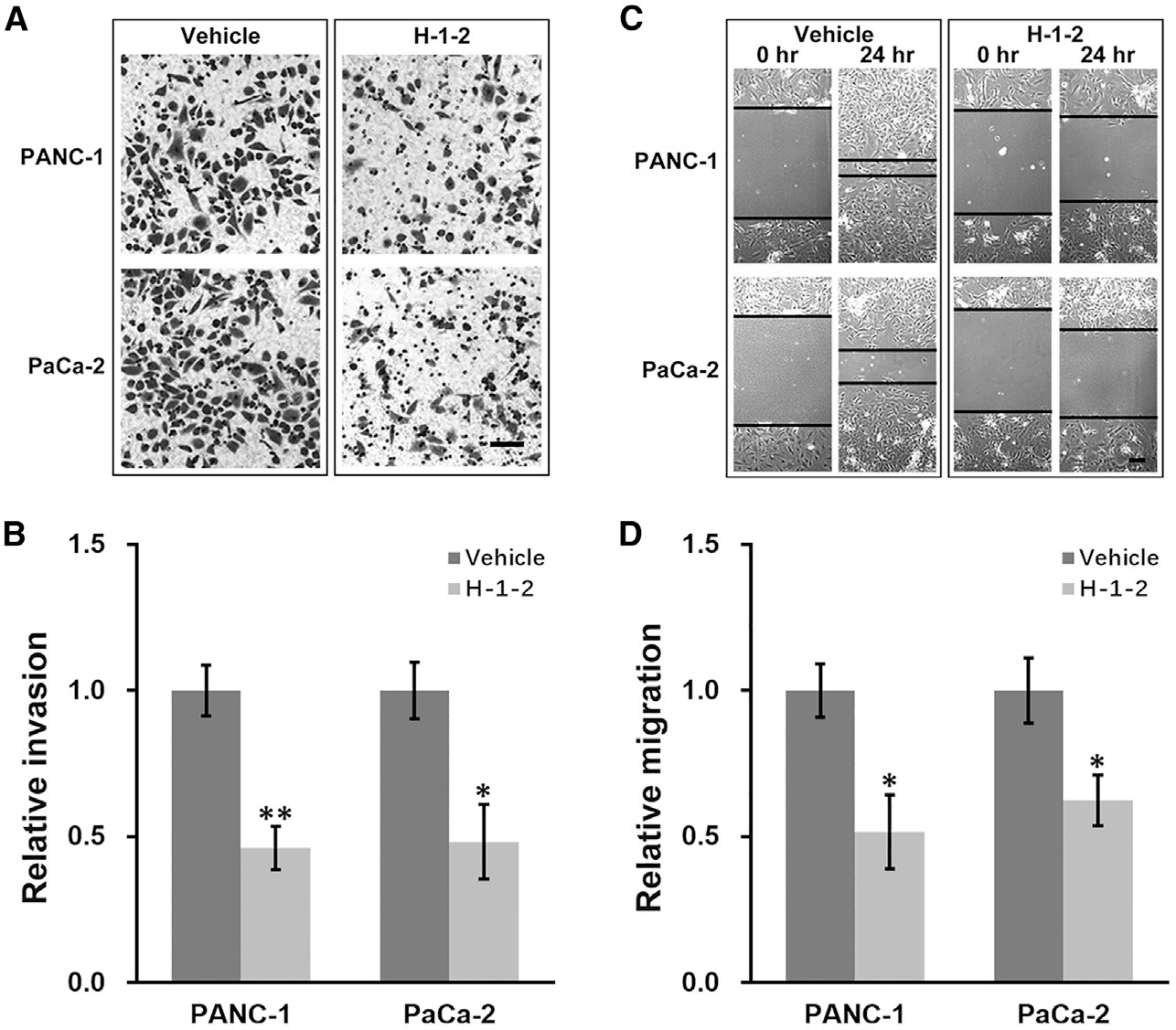
H-1-2 Inhibits Invasion and Migration of Pancreatic Cancer Cells *In Vitro* (A–D) PANC-1 and PaCa-2 cells were treated with vehicle or H-1-2, respectively, and subjected to transwell invasion (A and B) and scratch migration (C and D) assays. Representative images of both assays are shown in (A) and (C), with quantifications shown in (B) and (D), respectively. Data are means ± SD from at least three independent biological repeats. ∗p < 0.05, ∗∗p < 0.01, compared to vehicle.

To examine the effect of H-1-2 on invasion and migration of pancreatic cancer cells *in vitro*, we performed Transwell invasion and wound healing assays in both PANC-1 and PaCa-2 cells after treatments with vehicle or H-1-2, respectively. Invasiveness of both cell lines were significantly reduced by H-1-2 compared to the vehicle control (Figures 1A and 1B). Similarly, the same reduction trend was observed in the migration of both cell lines (Figures 1C and 1D). These findings indicated that *in vitro* growth of pancreatic cancer cells PANC-1 and PaCa-2 was suppressed by H-1-2.

### H-1-2 Inhibits Growth of Xenograft Pancreatic Tumors and Increases Survival of Mice Bearing Xenograft Tumors

**Figure 2:**
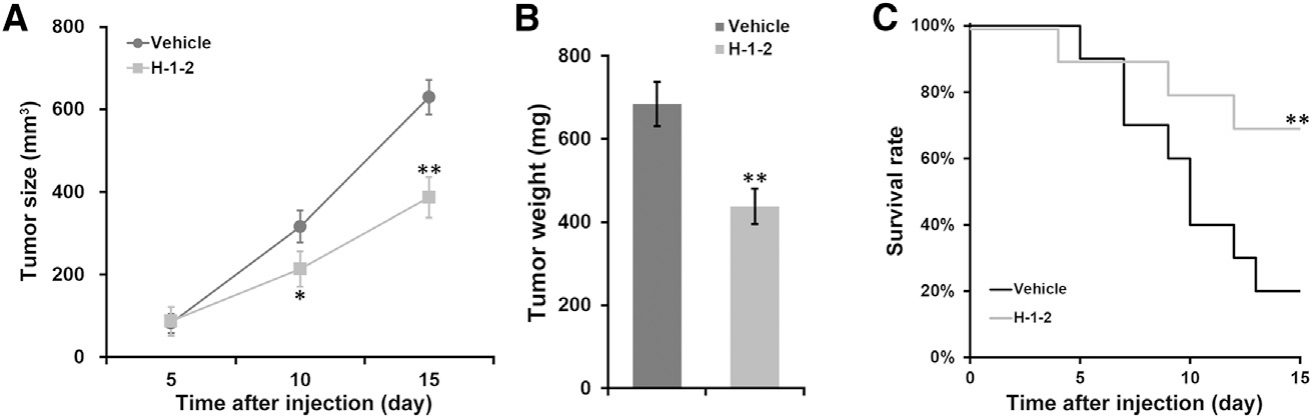
H-1-2 Inhibits Growth of Xenograft Pancreatic Tumors and Increases Survival of Mice Bearing Xenograft Tumors (A) Xenograft pancreatic tumor volume was monitored in mice receiving vehicle and H-1-2 treatment, respectively. (B) On day 15 after injection, mice were sacrificed to measure tumor weight. (C) Survival rates of mice receiving vehicle and H-1-2 treatment were monitored for up to 15 days. Data are means ± SD (n = 10 each). ∗p < 0.05, ∗∗p < 0.01, compared to vehicle.

Next, we sought to experimentally validate the above observation *in vivo* with a xenograft pancreatic tumor mouse model. Our results unambiguously demonstrated that administration of H-1-2 significantly suppressed xenograft tumor growth (Figure 2A), which firmly consolidated the anti-tumor property of H-1-2 *in vivo*. Consistently, the weight of xenograft tumor resected at the endpoint of the experiment manifested the remarkable reduction in the H-1-2-treated mice (Figure 2B). Our data for the first time uncovered the suppressive effect of H-1-2 on pancreatic cancer progression *in vivo*. Furthermore, Kaplan-Meier analysis unraveled the prominent therapeutic effect of H-1-2, which significantly prolonged the overall survival time of mice bearing xenograft tumors in comparison with the vehicle group (Figure 2C).

### HIF1α and Anterior Gradient 2 (AGR2) mRNA Are Upregulated in Pancreatic Cancer Patient Tumor Tissues

**Figure 3:**
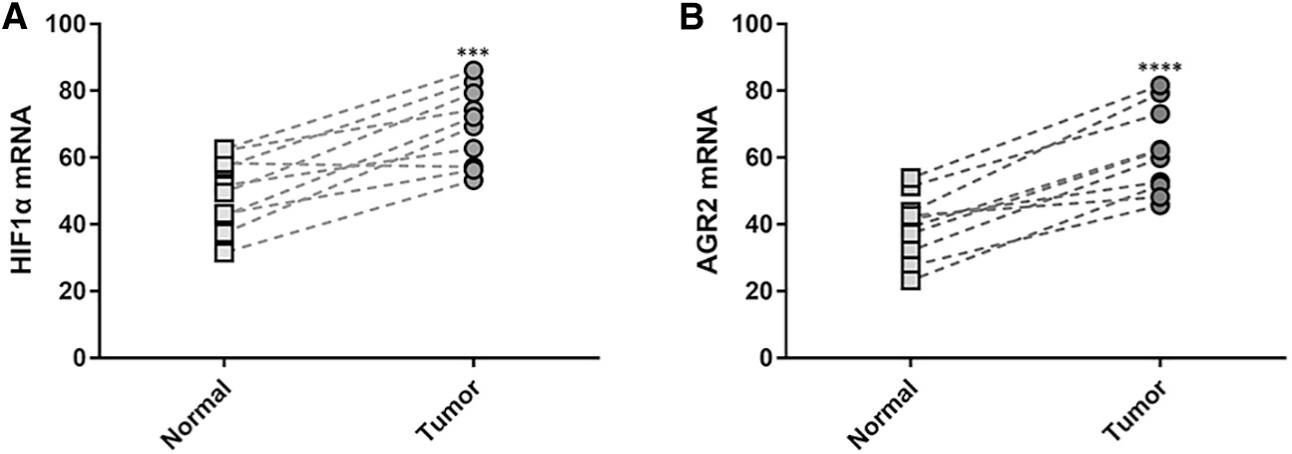
HIF1α and AGR2 mRNA Are Upregulated in Pancreatic Cancer Patient Tumor Tissues (A and B) mRNA levels of HIF1α (A) and AGR2 (B) were examined in pancreatic cancer patient tumor and adjacent normal tissues (n = 10). ∗∗∗p < 0.001, ∗∗∗∗p < 0.0001, compared to normal tissues.

We further investigated the potential downstream effectors responsible for the anti-tumor actions of H-1-2. HIF1α is a major regulator of cellular adaption to hypoxia and is enriched in most pancreatic cancer tissues. As expected, by comparing its expression level between pancreatic cancer patient tumor and adjacent normal tissues, we found HIF1α to be significantly upregulated in the patient tumors (Figure 3A). AGR2 was reported to be upregulated by HIF1α.^13^ We therefore reasoned that elevated HIF1α in pancreatic tumor tissues could lead to upregulated AGR2 expression as well, which was indeed confirmed by higher AGR2 mRNA levels in pancreatic tumor tissues than adjacent normal tissues (Figure 3B).

### H-1-2 Inhibits AGR2 and HIF1α in Pancreatic Cancer

**Figure 4:**
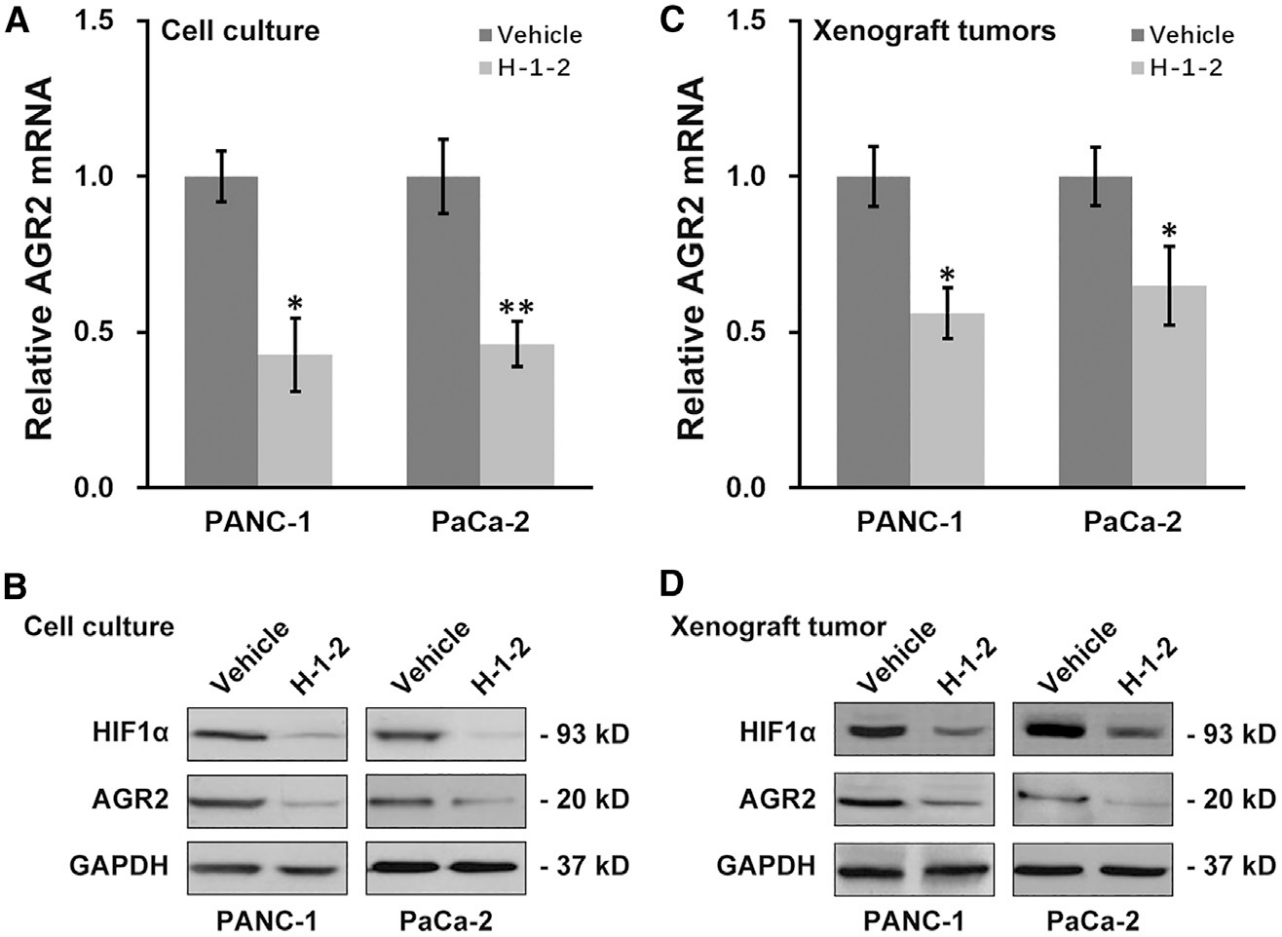
H-1-2 Inhibits AGR2 and HIF1α Expression in Pancreatic Cancer *In Vitro* and *In Vivo* (A and B) PANC-1 and PaCa-2 cells were treated with vehicle or H-1-2, respectively, followed by examination of AGR2 mRNA (A) and AGR2 and HIF1α protein (B) levels. Data are means ± SD from at least three independent experiments. (C and D) On day 15 after injection, xenograft pancreatic tumors were extracted from mice receiving vehicle and H-1-2 treatment, respectively, followed by examination of AGR2 mRNA (C) and AGR2 and HIF1α protein (D) levels. Data are means ± SD (n = 10 each). ∗p < 0.05, ∗∗p < 0.01, compared to vehicle.

Next, we sought to elucidate the molecular mechanism underlying the anti-tumor property of H-1-2 in pancreatic cancer. In view of the possible oncogenic contribution of AGR2 as indicated by our previous data, here we attempted to determine the relative expression of AGR2 in response to H-1-2 treatment. To this end, AGR2 mRNA was extracted from both pancreatic cancer cell lines and xenograft tumors after mice were sacrificed. Our quantitative real-time PCR results demonstrated that H-1-2 significantly repressed expression of AGR2 at the transcriptional level in both cell culture and xenograft tumors (Figures 4A and 4C). Consequently, AGR2 protein levels were also greatly reduced in the H-1-2-treated cell culture and in mice in comparison with vehicle control (Figures 4B and 4D). Notably, H-1-2 treatment also suppressed HIF1α protein levels (Figures 4B and 4D), confirming the previously reported anti-hypoxic property of H-1-2.^10^^,^^12^

### H-1-2 Downregulates AGR2 Expression through the Hypoxia-Responsive Element (HRE) in Its Promoter Region

**Figure 5:**
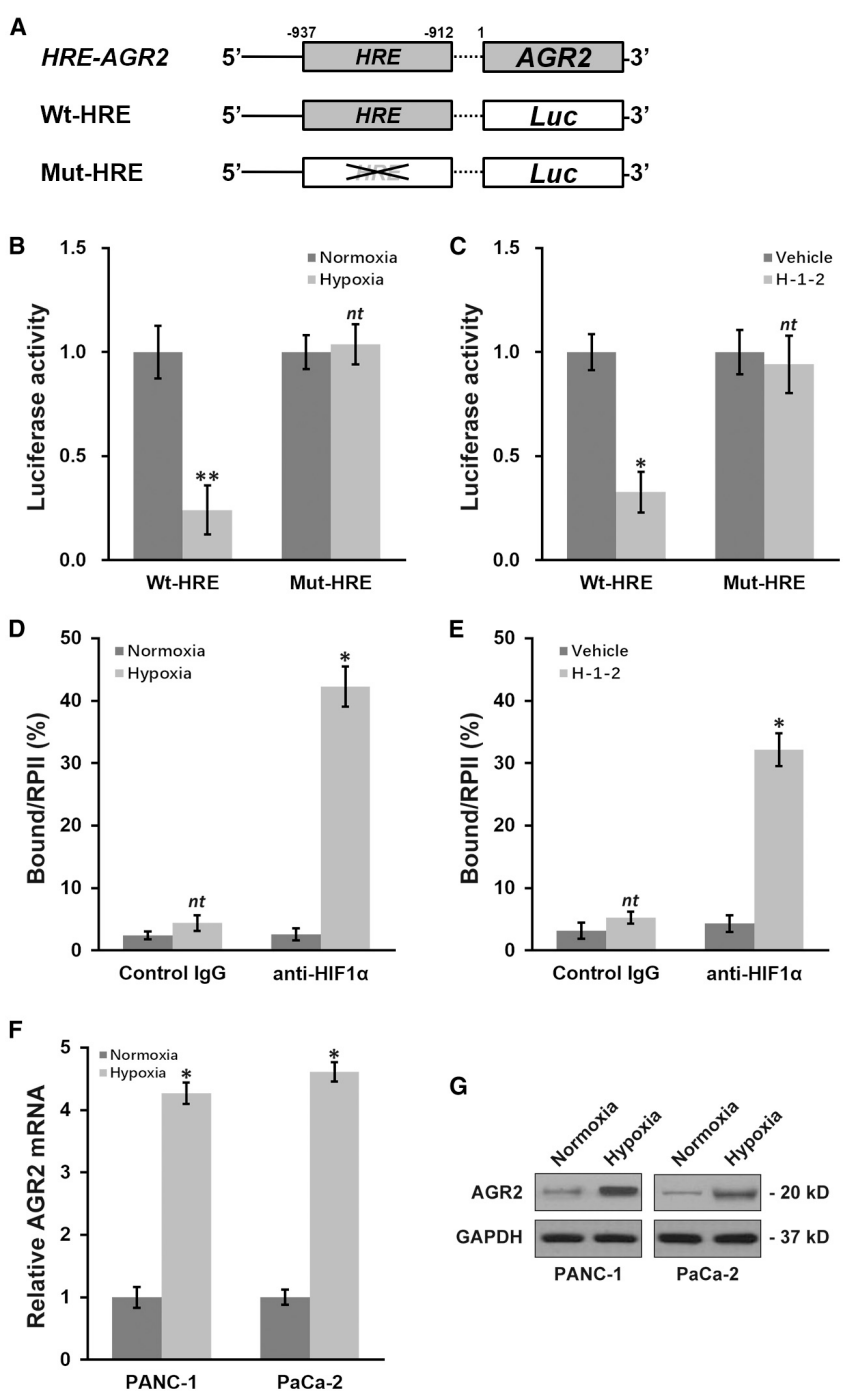
H-1-2 Downregulates AGR2 Expression through the Hypoxia Response Element (HRE) in Its Promoter Region (A) Promoter region of AGR2 contains a putative hypoxia response element (HRE). Wild-type (WT-HRE) or mutated (Mut-HRE) HRE sites from the AGR2 promoter were cloned upstream of a luciferase reporter gene open reading frame (Luc). (B) PANC-1 cells transfected with either WT-HRE or Mut-HRE constructs were subjected to normoxic (0 μM CoCl_2_) or hypoxic (100 μM CoCl_2_) conditions, respectively, followed by a luciferase reporter assay. (C) PANC-1 cells transfected with either WT-HRE or Mut-HRE constructs were treated with vehicle or H-1-2, respectively, followed by a luciferase reporter assay. (D) PANC-1 cells transfected with either WT-HRE or Mut-HRE constructs were subjected to normoxic (0 μM CoCl_2_) or hypoxic (100 μM CoCl_2_) conditions, respectively, followed by a ChIP assay using control immunoglobulin G (IgG) or anti-HIF1α antibody. (E) PANC-1 cells transfected with either WT-HRE or Mut-HRE constructs were treated with vehicle or H-1-2, respectively, followed by a ChIP assay using control IgG or anti-HIF1α antibody. (F and G) PANC-1 and PaCa-2 cells were subjected to normoxic (0 μM CoCl_2_) or hypoxic (100 μM CoCl_2_) conditions, respectively, followed by examination of AGR2 mRNA (F) and protein (G) levels. Data are means ± SD from at least three independent biological repeats. ^nt^p > 0.05, ∗p < 0.05, ∗∗p < 0.01, compared to normoxia or vehicle.

HIF1α was reported to promote AGR2 expression via the HRE from −937 to −912 bp on the AGR2 promoter sequence.^13^ To experimentally interrogate the modulation of AGR2 expression by HIF1α, we constructed wild-type or mutated HRE-fused luciferase reporter plasmids (Figure 5A). Under hypoxic conditions (100 μM CoCl_2_), luciferase activity of the wild-type construct was severely compromised compared to under normoxic conditions, whereas the mutated HRE construct was unaffected (Figure 5B). As expected, as H-1-2 was able to induce hypoxia, H-1-2 treatment elicited a significant inhibition on the luciferase activity of the wild-type, but not the mutated, construct, using the hypoxia condition as a positive control (Figure 5C). In addition, the direct binding of HIF1α onto the HRE of AGR2 was assessed using a chromatin immunoprecipitation (ChIP) assay, which revealed a marked enrichment of the AGR2 HRE segment in the HIF1α-immunoprecipitated complex under hypoxic conditions (Figure 5D). Similarly, H-1-2 treatment was also able to induce strong binding of HIF1α antibody to the AGR2 HRE (Figure 5E), showing a similar effect as the hypoxia positive control (Figure 5D). These findings clearly indicated that HIF1α directly bound to the HRE on AGR2 promoter to enhance its expression. It is also obvious that the anti-hypoxic property of H-1-2 was indeed responsible for its inhibitory effect on AGR2 expression, because both AGR2 mRNA and proteins were significantly upregulated by the hypoxic condition (100 μM CoCl_2_) in pancreatic cells (Figures 5F and 5G).

### Ectopic AGR2 Expression Partially Negates the H-1-2 Beneficial Effect against Pancreatic Cancer

**Figure 6:**
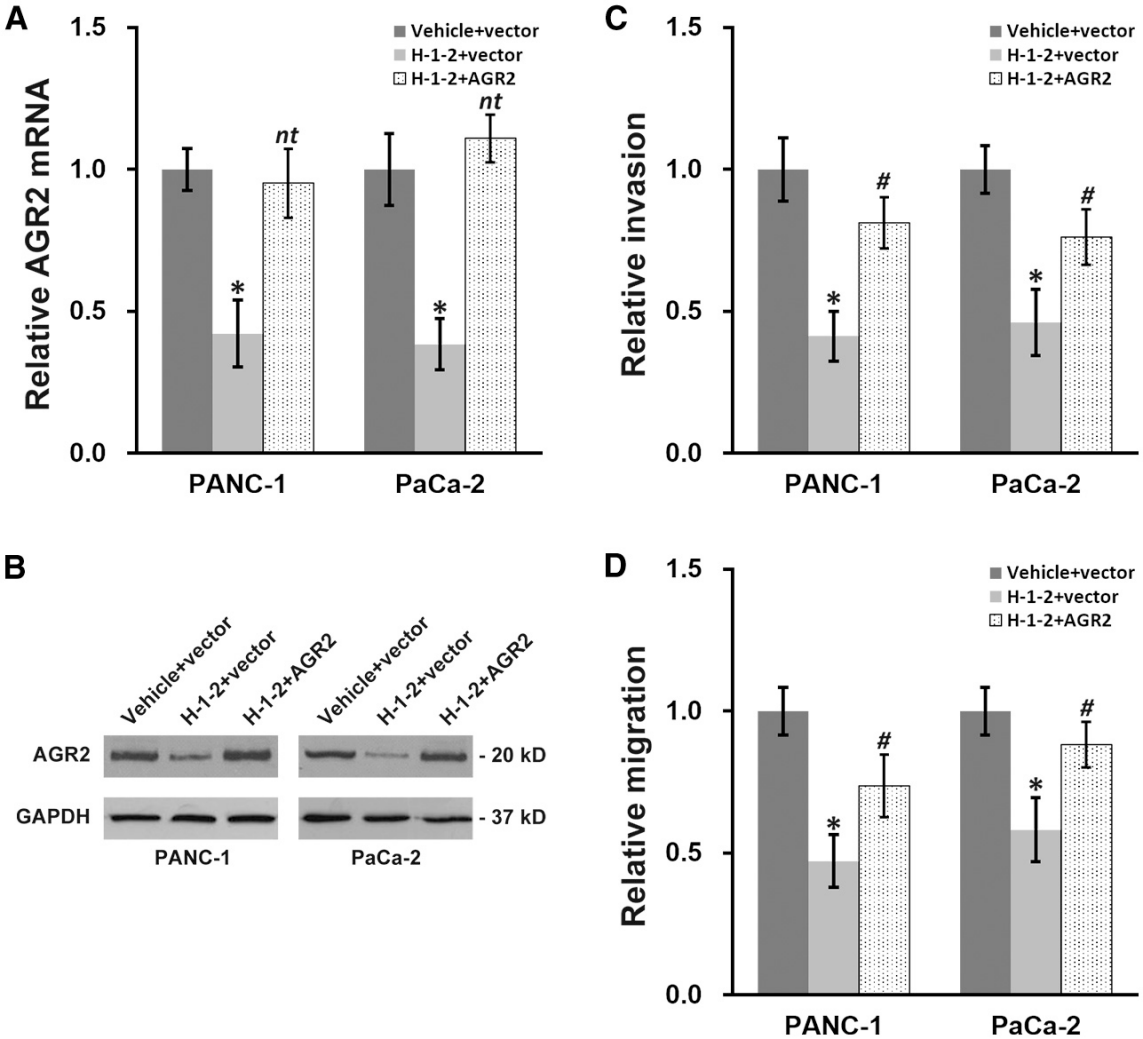
Ectopic AGR2 Expression Partially Negates an H-1-2 Inhibitory Effect on Pancreatic Cell Invasion and Migration PANC-1 and PaCa-2 cells expressing either vector control or AGR2 plasmid were also treated with vehicle or H-1-2, respectively. (A and B) Cells were subjected to examination of AGR2 mRNA (A) and protein (B) levels. (C and D) Cells were subjected to Transwell invasion (C) and scratch migration (C) assays. Data are means ± SD from at least three independent biological repeats. ^nt^p > 0.05, ∗p < 0.05, compared to both vehicle+vector and H-1-2+AGR2; ^#^p < 0.05, compared to vehicle+vector.

Our previous data demonstrated the anti-tumor property of H-1-2, which specifically inhibited AGR2 expression. Next, we sought to elucidate whether the repressed HIF1α expression played an essential role to mediate the anti-tumor effects of H-1-2. We introduced ectopic expression of AGR2 into H-1-2-treated pancreatic cells to antagonize the H-1-2-induced AGR2 inhibition, and this fully rescued AGR2 expression to comparable levels as for untreated cells (Figures 6A and 6B). We then examined the invasion and migration of these cells and discovered that the re-expression of AGR2 attenuated the inhibitory effects of H-1-2 on the *in vitro* growth of pancreatic cell lines, but only to a certain extent and not fully restored to levels seen in untreated cells (Figures 6C and 6D).

**Figure 7:**
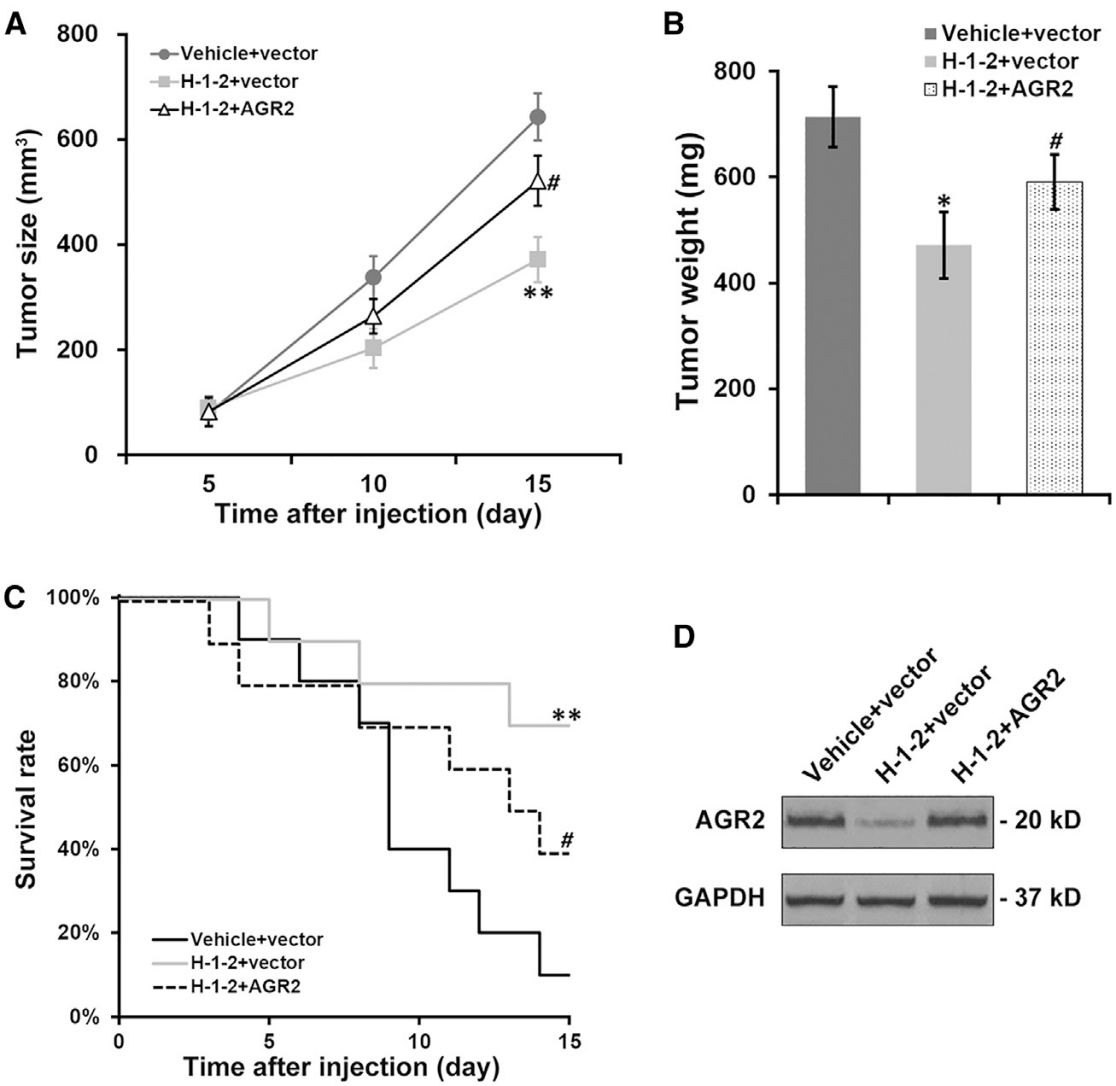
Ectopic AGR2 Expression Partially Negates H-1-2 Inhibitory Effect on Growth of Xenograft Pancreatic Tumors and H-1-2 Promotional Effect on Survival of Mice Bearing Xenograft Tumors (A) Volume of xenograft pancreatic tumors expressing either vector control or AGR2 plasmid was monitored in mice receiving vehicle and H-1-2 treatment, respectively. (B) On day 15 after injection, mice were sacrificed to measure tumor weight. (C) Survival rates of mice were monitored for up to 15 days. (D) On day 15 after injection, xenograft pancreatic tumors were extracted from mice, followed by examination of AGR2 protein levels. Data are means ± SD (n = 10 each). ∗p < 0.05, ∗∗p < 0.01, both vehicle+vector and H-1-2+AGR2; ^#^p < 0.05, compared to vehicle+vector.

Similarly, a partially compromised H-1-2 anti-tumor effect by AGR2 re-expression was also observed *in vivo*: ectopic introduction of AGR2 could restore the xenograft tumor growth (Figure 7A), and the weight of xenograft tumor at the endpoint was increased by re-expression of AGR2 in H-1-2-treated mice (Figure 7B), but not to control levels in both cases. Moreover, we observed significantly shortened survival of mice after injection with cells with re-introduced AGR2 even though mice were administered H-1-2, but the lifespans of these mice were nevertheless longer than those of control mice without H-1-2 treatment (Figure 7C). Finally, the extracted xenograft tumors at the endpoint were subjected to western blot analysis, which indicated complete rescue of AGR2 protein levels upon its ectopic expression in the H-1-2-treated group (Figure 7D).

## Discussion

**Figure 8:**
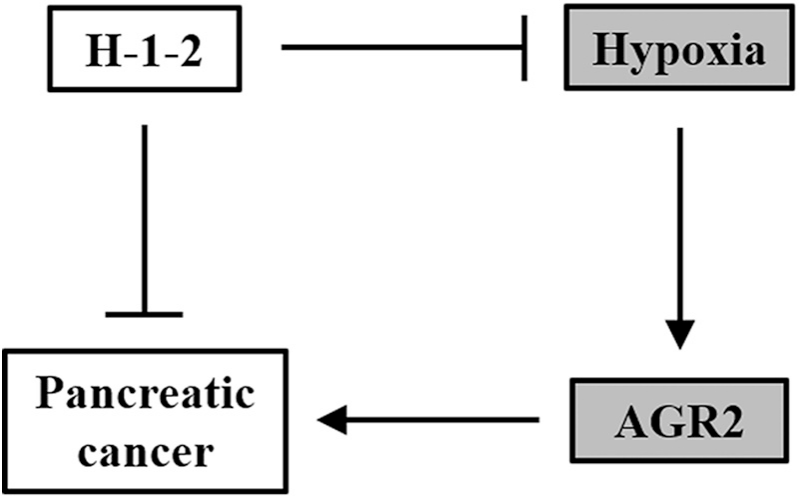
Schematic Illustration of the Working Model

In this study, we showed that treatment of the single-component polysaccharide H-1-2 was able to inhibit invasion and migration of pancreatic cancer cells *in vitro*, and xenograft pancreatic tumor growth *in vivo*, as well as to improve survival of mice bearing the xenograft pancreatic tumors. In searching for the underlying molecular effectors of H-1-2, we discovered that HIF1α and AGR2 mRNA were upregulated in tumor tissues of pancreatic cancer patients, both of which were previously implicated in the tumorigenesis of pancreatic cancer.^4^^,^^14^ Indeed, our results indicated that H-1-2 could inhibit AGR2 and HIF1α expression in pancreatic cancer both *in vitro* and *in vivo*. Through a luciferase reporter assay and ChIP, we have established a direct interaction between HIF1α protein and HRE on the promoter of AGR2 gene, which is responsible for the observed hypoxic induction of AGR2 expression. We therefore speculated that H-1-2 likely downregulated AGR2 expression through its anti-hypoxic property. To test this hypothesis, ectopic AGR2 expression was re-introduced into H-1-2-treated pancreatic cells, and this partially negated the H-1-2 inhibitory effect on cell invasion and migration abilities. Moreover, in the mouse model, ectopic AGR2 expression also negated the H-1-2 inhibitory effect on growth of xenograft pancreatic tumors and the H-1-2 promotional effect on survival of mice. These data clearly support the conclusion that H-1-2 suppresses pancreatic cancer by inhibiting hypoxia-induced AGR2 (Figure 8).

Anterior gradient (AG) genes were initially identified in *Xenopus laevis*,^15^ and AGR2 is one of the human homologs. Human AGR2 belongs to the protein disulfide isomerase (PDI) family of endoplasmic reticulum (ER)-resident proteins,^16^ with strong expression in the stomach, lung, prostate, small intestines, and colon.^17^ A structural relationship between the functional domains of AGR2 and the PDI family of molecular chaperones indicates potential AGR2 activity in protein folding.^18^ An increase in AGR2 levels has been reported in a variety of tumor tissues, but the molecular events resulting in the elevated AGR2 in tumor cells, as well as the clinical outcomes of AGR2 upregulation in tumors, remain largely unknown.^19^ Particularly in the context of pancreatic cancer, AGR2 was reported to be expressed and secreted during the progression of pancreatic cancer to promote the survival of cancer cells.^14^ Furthermore, as an ER stress protein, AGR2 reportedly preceded and contributed to the initiation of pancreatic cancer.^20^ In pancreatic intraepithelial neoplasia, AGR2 acts as a SMAD4-suppressible gene that regulates MUC1 levels and stimulates the initiation and progression of cancer.^21^ Importantly, an elevated level of AGR2 in pancreatic cancer tissues was verified using immunohistochemistry, cDNA, and tissue microarrays,^22^^,^^23^ while downregulation of AGR2 led to cell apoptosis and attenuated chemotherapy resistance of pancreatic cancer cells through the extracellular signal-regulated kinase (ERK)/AKT axis.^24^ These findings suggest that AGR2 promotes the survival of pancreatic cancer cells and endows tumor cells with the protection from chemotherapeutic treatments, which possibly contributes to the fact that pancreatic cancer is widely recognized as highly resistant to therapeutic interventions. Our results, where H-1-2 repressed AGR2 expression in pancreatic cancer cells through its anti-hypoxic function, provide yet another instance implicating AGR2 as an important prognosis factor for treatment against pancreatic cancer.

Clinical studies have clearly demonstrated that hypoxia is a common feature shared by various solid tumors. Normal tissues are generally under an oxygen (O_2_) pressure of 30–50 mm Hg, which decreases to below 2.5 mm Hg in up to half of locally advanced solid tumors.^25^ Measurement of O_2_ levels using pO_2_ histography in patients with several solid tumors revealed that pancreatic cancer was associated with the most hypoxic conditions.^26^ Similarly, intraoperative pO_2_ measurements of seven resectable pancreatic cancer tissues further confirmed the hypoxic microenvironment.^27^ Under normoxic conditions, HIF1α is constitutively hydroxylated by prolyl hydroxylase^28^ and subsequently ubiquitinated by the von Hippel-Lindau (VHL) gene,^29^ which results in the degradation via the ubiquitin-proteasome system. In response to the reduced oxygen supply, HIF1α is stabilized, accumulated, and forms heterodimers with HIF1β to transcriptionally activate various downstream genes.^30^ In the context of this study, H-1-2 exerts its beneficial role against pancreatic cancer by targeting hypoxia/HIF1α, supporting HIF1α as a promising therapeutic target in pancreatic cancer treatments.

Hypoxic regulation of AGR2 in tumor biology has been previously documented. For instance, in glioblastoma, AGR2 was increased by HIF1, which led to enhanced migration and tube formation capabilities of cells *in vitro* and increased growth and vascularity of tumor xenografts *in vivo*.^31^ Alternatively, AGR2 was regarded as a binding stabilizer of HIF1α, thereby contributing to the hypoxia-induced doxorubicin resistance in breast cancer.^13^ However, no prior investigation has been conducted on the role of hypoxia/AGR2 regulation in pancreatic cancer. In line with this, our current study is the first instance to provide evidence demonstrating the direct involvement of hypoxia-regulated AGR2 in tumorigenesis of human pancreatic cancer. Using a luciferase reporter assay, we have shown that the −937- to −912-bp promoter region upstream of the AGR2 open reading frame is an HRE. In addition, this HRE was able to directly recruit HIF1α binding in a ChIP assay.

Notably, although ectopic expression of AGR2 almost fully rescued its expression in H-1-2-treated pancreatic cancer cells to comparable level as untreated cells, it did not completely abolish the beneficial effect of H-1-2 on pancreatic cancer cells. Instead, it only partially restored the invasion and migration abilities *in vitro* and xenograft tumor growth *in vivo*. This observation raises the possibility that, besides AGR2, there are likely other downstream targets of HIF1α participating in the tumorigenesis of pancreatic cancer, which calls for further investigations.

### Conclusion

To conclude, we have demonstrated a beneficial therapeutic effect of polysaccharide H-1-2, a bioactive component of *P. heterophylla*, against pancreatic cancer both *in vitro* and *in vivo*. H-1-2 inhibits hypoxia, which consequently downregulates AGR2 expression, to suppress pancreatic tumor. Our current study supports further investigation into the efficacy of H-1-2 against pancreatic cancer in clinical settings. This study is, however, limited in the following aspects: (1) the relatively small number of clinical samples, and (2) the observed mechanism needs to be verified in clinic.

## Materials and Methods

### Cell Lines

Two pancreatic cell lines, PaCa-2 and PANC-1, were obtained from the American Type Culture Collection (ATCC, Manassas, VA, USA) and cultured in Dulbecco’s modified Eagle’s medium (DMEM)/F12 (Sigma-Aldrich, St. Louis, MO, USA) supplemented with 1% penicillin-streptomycin (Gibco, Grand Island, NY, USA) and 10% fetal bovine serum (FBS; Invitrogen, Waltham, MA, USA) in a humidified incubator supplied with 5% CO_2_ at 37°C. Both cell lines were verified using the short tandem repeat analysis. H-1-2 was purified with purity >95% and prepared in culture media to a final concentration of 100 μg/mL according to a previous report.^11^

### Transwell Invasion Assay

Transwell invasion assay was performed using inserts of 8-μm pore size (BD Biosciences, Franklin Lakes, NJ, USA) inside a 24-well plate. Cells at a density of 1 × 10^5^ cells per well were seeded into the upper compartment containing the Matrigel-coated membrane, then 500 μL of medium supplemented with 10% FBS was added to the lower compartment to engage cells. Next, cultures were maintained in a 5% CO_2_ humidified incubator at 37°C for 24 h, followed by treatment with 20 μM 5-ethynyl-2′-deoxyuridine at 37°C for another 4 h. After that, inserts were detached to be visualized using the ENU kit (Invitrogen, Waltham, MA, USA). To calculate the invasion rate, cells were quantified from six randomly selected fields for each individual well and normalized to the appropriate control.

### Scratch Migration Assay

Cells were cultured in six-well plates and allowed to reach 100% confluence, and then starved in growth media without FBS in the presence of 10 μg/mL mitomycin C (Sigma-Aldrich, St. Louis, MO, USA). For the wound-healing assay, a linear incision was made across the cell monolayer with the tip of a 100-μL sterile pipette, and cultures were subsequently washed with phosphate-buffered saline (PBS) to remove the debris. Cells were cultured in the presence of 10 μg/mL mitomycin C for another 24 h. The distance that the wound edge moved was measured to calculate the migration rate, which was normalized to the appropriate control.

### Xenograft Mouse Model

The NSG immunodeficient mice were obtained from Biocytogen (Beijing, China) and allowed to acclimate for at least 1 week before experiments. Mice were housed in the specific pathogen-free (SPF) environment with free access to feed and sterile drinking water. All animal experiments were executed in conformity with the *Guide for the Care and Use of Laboratory Animals* by the NIH. The protocols and experimental design were approved by the Animal Ethics Committee of The First Affiliated Hospital of Wenzhou Medical University. To establish the xenograft mouse model, 10^6^ cells in 100 μL of vehicle were subcutaneously inoculated into the lower flank of the animal. The growth of the tumor was regularly monitored and recorded. H-1-2 (1.5 g/kg body weight) was delivered via oral gavage for 30 consecutive days and controlled by drinking water as previously established.^32^ No side effects were observed in animals administered with the indicated dose of H-1-2.

### Patient Samples

The clinical samples were collected from 10 pancreatic cancer patients in The First Affiliated Hospital of Wenzhou Medical University, and pathologically was confirmed by three experts independently. Pancreatic cancer tissues, together with paired adjacent normal tissues, were harvested during surgical procedures and then immediately snap-frozen with liquid nitrogen for further analysis. The protocols were approved and authorized by the Ethics Committee of The First Affiliated Hospital of Wenzhou Medical University. All enrolled patients provided written informed consents.

### Quantitative Real-Time PCR

Total RNA of the exponential cells was extracted with a TRIzol reagent kit (Invitrogen, Waltham, MA, USA). The concentration of RNA was assessed using the NanoDrop 1000 (Thermo Fisher Scientific, Waltham, MA, USA), and the integrity of RNA samples was examined through agarose electrophoresis. Preparation of cDNA was immediately performed using a high-capacity cDNA reverse transcription kit (Thermo Fisher Scientific, Waltham, MA, USA) with mixed oligo(dT) and random primers. Quantitative real-time PCR was conducted using SYBR Green real-time PCR master mixes (Thermo Fisher Scientific, Waltham, MA, USA), and the relative expression of genes was calculated using the 2^−ΔΔCt^ method. Primers used in the current study were as follows: HIF1α forward 5′-AGGTGGATATGTCTGGGT-3′, reverse 5′-AAGGACACATTCTGTTTGTTG-3′; AGR2 forward 5′-GGAGGACAAACTGCTCTGCCAA-3′, reverse 5′-TCCAAGACAACAAACCCTTG-3′; GAPDH forward 5′-CTGACTTCAACAGCGACACC-3′, reverse 5′-TAGCCAAATTCGTTGTCATAC-3′.

### Western Blot

Cell lysate was prepared in standard radioimmunoprecipitation assay (RIPA) lysis buffer and the protein content was quantified using the bicinchoninic acid (BCA) protein assay kit (Thermo Fisher Scientific, Waltham, MA, USA). Samples of equal amounts of protein were resolved using sodium dodecyl sulfate-polyacrylamide gel electrophoresis and subsequently transferred onto polyvinylidene fluoride membrane on ice. The membrane was then blocked with 5% skim milk in Tris-buffered saline with 0.1% Tween 20 (TBST) buffer on a shaker at room temperature for 1 h and subjected to incubation with the primary antibodies (anti-HIF1α, Cell Signaling Technology, 1:1,000; anti-AGR2, Novus Biologicals, 1:250; anti-GAPDH, Abcam, 1:1,000) overnight at 4°C. After six washes with TBST (5 min each), the membrane was blotted with the appropriate secondary antibody at room temperature for 1 h and then visualized using a commercial enhanced chemiluminescence (ECL) kit (Millipore, Billerica, MA, USA). GAPDH was used as the internal loading control.

### Luciferase Reporter Assay

The promoter region of AGR2 including the putative HRE was subcloned into the pGL3 luciferase reporter plasmid for transient transfection using Lipofectamine 2000. The recipient cells were exposed to either normoxic (0 μM CoCl_2_) or hypoxic (100 μM CoCl_2_) conditions. The relative luciferase activities were examined 24 h after transfection using the commercially available Bright-Glo luciferase reporter system (Promega, Madison, WI, USA).

### ChIP

The ChIP assay was conducted using commercially available kits obtained from Abcam (Ab500, Cambridge, MA, USA) following the manufacturer’s instructions. In brief, the exponential cells exposed to hypoxia-mimicking stimulations were first crosslinked with 1% formaldehyde at room temperature for 20 min and then transferred to lysis buffer on ice for 10 min. The cell lysate was centrifuged, the supernatant was discarded, and the chromatin pellet was re-suspended and subjected to ultrasonic shearing with three 15-s pulses with one 30-s interval of rest on ice in between. The target chromatin fragments were immunoprecipitated using HIF1α antibody overnight at 4°C and released through incubation with RNase and proteinase K in DNA release buffer for 30 min at 42°C. The enrichment of candidate promoters was evaluated by subsequent PCR.

### Ectopic AGR2 Expression

The full-length cDNA sequence of the human AGR2 open reading frame was PCR amplified and cloned into the pcDNA3.1 vector (Life Technologies, Pleasanton, CA, USA), using standard molecular cloning protocol.

### Statistical Analysis

The results were obtained from three independent biological repeats unless otherwise specified and presented as mean ± standard deviation (SD). Data were analyzed using GraphPad Prism 7.0 software. The one-way ANOVA followed by a Tukey test was performed for statistical comparison. p < 0.05 was considered statistically significant.

## Author Contributions

H.S., K.S., K.Q., H.K., and Q.H. conducted the experiments; M.Z. designed the study; and H.S., K.S., K.Q., and M.Z. wrote the manuscript.

## Conflicts of Interest

The authors declare no competing interests.
